# Inferring the Probability of the Derived *vs.* the Ancestral Allelic State at a Polymorphic Site

**DOI:** 10.1534/genetics.118.301120

**Published:** 2018-05-16

**Authors:** Peter D. Keightley, Benjamin C. Jackson

**Affiliations:** Institute of Evolutionary Biology, School of Biological Sciences, University of Edinburgh, EH9 3FL, United Kingdom

**Keywords:** nucleotide polymorphism, ancestral allele, derived allele, unfolded site frequency spectrum, parsimony, misinference

## Abstract

It is known that the allele ancestral to the variation at a polymorphic site cannot be assigned with certainty, and that the most frequently used method to assign the ancestral state—maximum parsimony—is prone to misinference. Estimates of counts of sites that have a certain number of copies of the derived allele in a sample (the unfolded site frequency spectrum, uSFS) made by parsimony are therefore also biased. We previously developed a maximum likelihood method to estimate the uSFS for a focal species using information from two outgroups while assuming simple models of nucleotide substitution. Here, we extend this approach to allow multiple outgroups (implemented for three outgroups), potentially any phylogenetic tree topology, and more complex models of nucleotide substitution. We find, however, that two outgroups and the Kimura two-parameter model are adequate for uSFS inference in most cases. We show that using parsimony to infer the ancestral state at a specific site seriously breaks down in two situations. The first is where the outgroups provide no information about the ancestral state of variation in the focal species. In this case, nucleotide variation will be underestimated if such sites are excluded. The second is where the minor allele in the focal species agrees with the allelic state of the outgroups. In this situation, parsimony tends to overestimate the probability of the major allele being derived, because it fails to account for the fact that sites with a high frequency of the derived allele tend to be rare. We present a method that corrects this deficiency and is capable of providing nearly unbiased estimates of ancestral state probabilities on a site-by-site basis and the uSFS.

MANY population genetic and quantitative genetic analysis methods require the assignment of ancestral *vs.* derived states at polymorphic nucleotide sites. For example, Fay and Wu (2000) and Zeng *et al.* (2006) proposed statistics, *H* and *E*, that compare the numbers of high, intermediate, and low frequency derived variants, which can then be used to distinguish between different modes of natural selection and demographic change. A number of methods have also been developed to infer selection and demographic change based on the complete distribution of counts of derived alleles across sites, the unfolded site frequency spectrum (uSFS) (*e.g.*, Boyko *et al.* 2008; Schneider *et al.* 2011; Tataru *et al.* 2017).

The minor allele at a site or counts of numbers of minor alleles at a group of sites (the folded site frequency spectrum) can be observed directly from sequence polymorphism data. In contrast, the derived *vs.* the ancestral allele at a site cannot be known with certainty, because at least one outgroup is required for inference, and there is the possibility of more than one mutation separating the focal species from the outgroup. This also implies that the uSFS cannot be known precisely. For the purpose of ancestral state inference, rule-based maximum parsimony is the most frequently applied method in molecular evolutionary genetics (*e.g.*, Voight *et al.* 2006; Dreszer *et al.* 2007; Keinan *et al.* 2007; Sabeti *et al.* 2007; 1000 Genomes Project Consortium 2010, 2015; Lohse and Barton 2011; Langley *et al.* 2012; Schmidt *et al.* 2017). It has been recognized, however, that parsimony potentially produces misleading results (Felsenstein 1981; Collins *et al.* 1994; Eyre-Walker 1998). Of particular relevance here is that sites that have a low frequency of the derived allele are usually more common than sites that have a high frequency of the derived allele. This implies that misinference tends to upwardly bias counts of high frequency derived alleles (Baudry and Depaulis 2003; Hernandez *et al.* 2007).

There is a related problem concerning the assignment of ancestral states, which does not seem to have been addressed. If ancestral states are assigned on site-by-site basis, potentially useful information is ignored. For example, consider the case of a single outgroup species that is uninformative about the ancestral allele of the variation in a focal species at a site. It is more likely, however, that the ancestral allele at the site is the low frequency allele, if sites with a high frequency of the derived allele are uncommon in the data set as a whole (as is usually the case).

Matsumoto *et al.* (2015) pointed out that ancestral states are not observable, that a single best ancestral reconstruction is not advisable, and that assuming one can bias molecular evolutionary inference. This was developed by Jackson *et al.* (2017), who assigned the ancestral state probability at a site as the inferred probability of the node for the common ancestor of the focal species and the closest outgroup, obtained using PAML (Yang 2007), while ignoring polymorphism data. However, this does not optimally weight information coming from the focal site itself and from the data as a whole.

Inference of ancestral states on a site-by-site basis has been problematic, but there has been progress in inferring the uSFS. Hernandez *et al.* (2007) developed a context-dependent substitution model using a single outgroup to infer the ancestral state at a polymorphic site in a focal species, and then implemented a step to correct for ancestral misidentification. Their simulations suggested, however, that the approach only partially corrects for ancestral misidentification, depending on the divergence between the focal species and the outgroup.

Schneider *et al.* (2011) developed a probabilistic method to infer the uSFS on a site-by-site basis, but did not use information from the frequencies of polymorphisms across all sites, so results from this method are biased. Keightley *et al.* (2016) developed a maximum likelihood (ML) method that addresses the deficiency in Schneider *et al.* (2011) and simulations suggested that it is capable of correctly inferring the uSFS. It uses a two-stage process in which the evolutionary rates are estimated by ML and then, assuming the rates, estimates the uSFS elements by ML, while correctly weighting information from informative and uninformative sites. However, the method is limited to two outgroups, assumes simple substitution models [for one outgroup, the Kimura two-parameter (K2) model; for two outgroups, the Jukes–Cantor (JC) model], and is not readily scalable to more than two outgroups or to more complex substitution models. It is unknown whether more realistic substitution models and/or additional outgroups significantly improves inference accuracy. Furthermore, it does not assign ancestral state probabilities on a site-by-site basis.

In this article, we develop the approach of Keightley *et al.* (2016), with the following objectives: (1) estimate the uSFS, allowing several outgroups, potentially any tree topology, and more realistic nucleotide substitution models; and (2) infer ancestral state probabilities for each polymorphic site in the data. We evaluate the performance of the new approach by simulations, apply it to data from the *Drosophila* Population Genomics Project (DPGP) as a test case, and reinfer the ancestral state probabilities for a population of the 1000 Genomes Project in humans, which were previously inferred by a parsimony-related approach.

## Materials and Methods

Following Keightley *et al.* (2016), uSFS inference is carried out in two-steps. Evolutionary rate parameters are estimated from all sites in the data (including polymorphic and monomorphic sites) in step 1. In step 2 the uSFS is computed, conditional on the evolutionary rate parameter estimates. Information from steps 1 and 2 is then combined in a third step to infer the ancestral state probability for each polymorphic site.

### Representation of the data and some definitions

**Figure 1:**
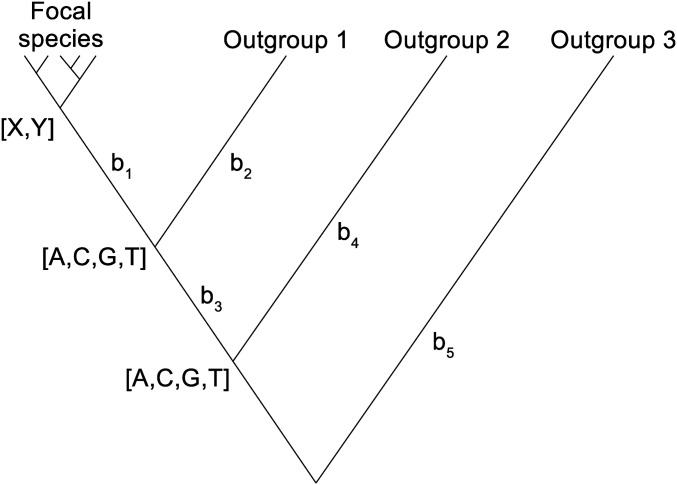
Representation of the data for uSFS and ancestral state inference. Polymorphism within the focal species (nucleotides X, Y) is assumed to coalesce within branch b_1_. There are three outgroups, two unknown internal nodes, and five branches in this tree. The root of the tree is not identifiable, therefore branch b_5_ extends from outgroup 3 to the node of b_3_ and b_4_.

Suppose we have sampled *m* orthologous gene copies at a set of sites from a population of a focal species. The uSFS we require to estimate therefore contains *m* − 1 elements, excluding the elements where the ancestral or derived allele is fixed. We assume that we have randomly sampled a single gene copy at each site in one or more outgroup species. We assume that the tree topology relating the species is known and does not vary among sites (Figure 1). In the analysis we assume that the nucleotide variation within the focal species coalesces within the branch labeled b_1_. The consequences of polymorphism in the outgroup species and violation of the assumptions of an invariant tree topology and coalescence within branch b_1_ are investigated in simulations. The observed nucleotide configuration for a site is the count of each of the four nucleotides in the focal species (labeled X, Y for a biallelic site), along with the state for each outgroup (A, C, G, or T). Let the number of outgroups = *n* (in Figure 1, *n* = 3), and denote the outgroups *o*_1_, *o*_2_...*o_n_*. Assuming an unrooted tree (as in Figure 1), the number of branches in the tree is therefore *b* = 2*n* − 1.

### Models of nucleotide substitution

**Figure 2:**
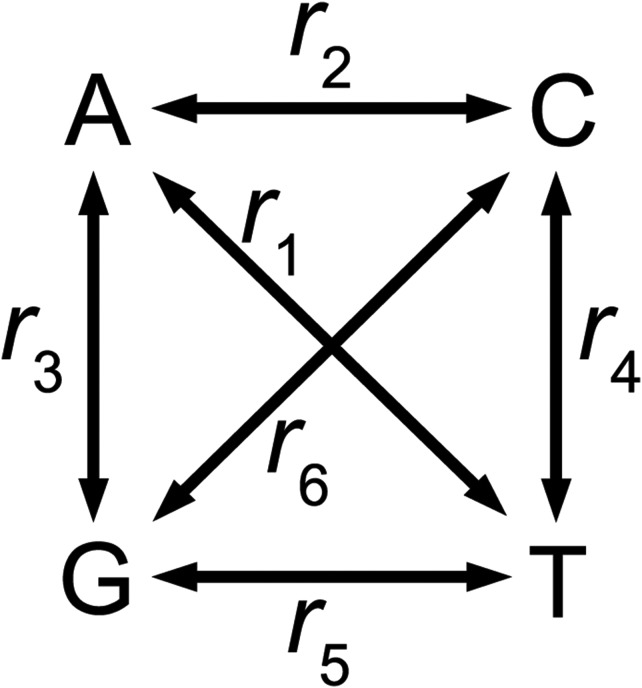
The R6 model.

The JC model, K2 model, and a model allowing six symmetrical rates (R6; Figure 2) are considered. All substitution models require the estimation of evolutionary rates (*i.e.*, mean number of nucleotide changes per site) for each branch, *K*_1_...*K_b_*. The rates are the only parameters for the JC model. For the K2 model, an additional parameter, *κ*, specifies the rate of transition mutations relative to the rate of transversions. For the R6 model, there are six symmetrical relative mutation rates, *r*_1_...*r*_6_,$\sum_{i=1}^{6}r_{i}=1$(Figure 2), so five independent parameters, *r*_1_...*r*_5_, require to be estimated.

### Estimation of rate parameters

Assuming the tree topology of Figure 1, there are *b* substitution rates and these, along with parameters of the substitution model (*i.e.*, *κ* for the K2 model or *r*_1_...*r*_5_ for the R6 model), are estimated by ML using the simplex algorithm for likelihood maximization. We checked convergence by picking starting values for the parameters from wide distributions, restarting the algorithm when convergence had apparently been achieved, and checking that the same final maximum log likelihood was reached in multiple runs. Let **ϕ** be a vector specifying the model parameters, and let **y**_i_ be a vector specifying the observed nucleotide configuration for the focal species and the outgroups at site *i*. Sites are assumed to evolve independently, so the overall likelihood of the data is the product of probabilities of the observed nucleotide configuration for each site:

$$L=\prod_{i=1}^{\text{sites}}p(y_{i}|\phi ).$$ (1)

The probability of the nucleotide configuration for each site is evaluated by summing the probabilities for the *n*_tree_ = 4*^n^* ^− 1^ possible unrooted trees, formed from all possible nucleotide combinations [A, T, G, C] at the unknown internal nodes along with the observed nucleotide configuration for the focal species and outgroups at the site.

$$p\left(y_{i}|\phi \right)=\sum_{j=1}^{n_{\text{tree}}}p_{\text{tree}}\left(c_{j}|\phi \right),$$ (2)

where **c_j_** is a vector representing the observed nucleotide configuration for the focal species and the *n* outgroups along with the nucleotide states for the *b* − 1 internal nodes for tree *j*. If the focal species is polymorphic at a site, the probability for that site is computed as the average probability for each observed nucleotide (X, Y in Figure 1).

The overall probability for a given tree is computed from the product of the probabilities of each branch (*k* = 1...*b*), conditional on the nucleotide states *x*_1,_*_k_* and *x*_2,_*_k_* representing the ancestral and derived nucleotides of that branch, given the nucleotide states specified in **c_j_**:

$$p_{\text{tree}}\left(c_{j}|\phi \right)=\prod_{k=1}^{n_{B}}p_{\text{branch}}\left(x_{1,k},x_{2,k}|\phi \right).$$ (3)

The probability for a branch depends on whether *x*_1,_*_k_* and *x*_2,_*_k_* differ from one another, the type of any difference (except in the case of the JC model), and the substitution rate parameters **ϕ**.

### Computation of *p*_branch_

In computing the probability of observing nucleotides *x*_1,_*_k_* and *x*_2,_*_k_* on branch *k*, it is assumed that the number of nucleotide changes on the branch is Poisson distributed. Terms for more than two changes on a branch are disregarded. The method could be extended to allow more than two changes on a branch, but highly saturated sites would contribute little useful information. Let *K_k_* be the evolutionary rate parameter for branch *k*, which is the mean number of changes for that branch.

#### JC model:

$$1.\;x_{1,k}=x_{2,k}:p_{\text{branch}}=\exp\left(-K_{k}\right)+\frac{1}{6}K_{k}^{2}\exp\left(-K_{k}\right)$$ (4)

$$2.\;x_{1,k}\neq x_{2,k}:p_{\text{branch}}=\frac{1}{3}K_{k}\exp\left(-K_{k}\right)+\frac{1}{9}K_{k}^{2}\exp\left(-K_{k}\right)$$ (5)

#### K2 model:

$$1.\;x_{1,k}=x_{2,k}:p_{\text{branch}}=\exp\left(-K_{k}\right)+\frac{1}{2}K_{k}^{2}\exp\left(-K_{k}\right)\frac{2+\kappa^{2}}{\kappa^{2}+4\kappa +4}$$ (6)

2. *x*_1,_*_k_* ≠ *x*_2,_*_k_*, transition change:

$$p_{\text{branch}}=K_{k}\exp\left(-K_{k}\right)\frac{\kappa }{\kappa +2}+K_{k}^{2}\exp\left(-K_{k}\right)\frac{1}{\kappa^{2}+4\kappa +4}\;$$ (7)

3. *x*_1,_*_k_* ≠ *x*_2,_*_k_*, transversion change:

$$p_{\text{branch}}=K_{k}\exp\left(-K_{k}\right)\frac{1}{\kappa +2}+K_{k}^{2}\exp\left(-K_{k}\right)\frac{\kappa }{\kappa^{2}+4\kappa +4}$$ (8)

#### R6 model (Figure 2):

$$1.\;x_{1,k}=x_{2,k}:p_{\text{branch}}=p\left(\text{0}\;\text{changes}\right)+p\left(\text{2}\;\text{changes}\right)$$ (9)

Taking the example of *x*_1,_*_k_* = *x*_2,_*_k_* = A:

$$p\left(\text{0}\;\text{changes}\right)=\exp\left[-2K_{k}\left(r_{1}+r_{2}+r_{3}\right)\right]$$ (10)

Note that *r*_1_, *r*_2_, and *r*_3_ are the relative rates for changes involving base A.

For *p*(2 changes): The algorithm to compute the probability of observing the same ancestral and derived base when two changes have occurred on a branch is illustrated by a simplified example where all relative rates in the model apart from two (*r*_1_ and *r*_4_) are zero (Figure 2).

For the case of *x*_1,_*_k_* = *x*_2,_*_k_* = A, the sequence of events must therefore be an A → T change followed by a T → A change. The probability of these events is obtained from:

$$\overset{1}{\underset{0}{\int }}p(\text{no mutation to time }y)\cdot p(\text{A}\to \text{T}\;\text{mutation})\cdot p(\text{T}\to \text{A}\;\text{mutation between time }y\text{ and}\;1)dy.$$ (11)

For the example where all relative rates in the model apart from *r*_1_ and *r*_4_ are zero, this is:

$$\frac{r_{1}^{2}}{\left(r_{1}+r_{2}+r_{3}\right)\left(r_{1}+r_{4}+r_{5}\right)}\overset{1}{\underset{0}{\int }}\text{exp}\left(-k_{1}y\right)k_{1}\left(1-y\right)k_{2}\times \exp\left[-k_{2}(1-y)\right]dy,$$ (12)

where *k*_1_ = 2*K_k_*(*r*_1_ + *r*_2_ + *r*_3_) and *k*_2_ = 2*K_k_*(*r*_1_ + *r*_4_ + *r*_5_). In this example, the relative rates *r*_2_, *r*_3_, and *r*_5_ are all zero, but are included for completeness. Evaluation of the definite integral in (12) gives a closed form expression:

$$\frac{k_{1}k_{2}\text{exp}(-k_{2}-k_{1})[\text{exp}(k_{2})-\text{exp}(k_{1})k_{2}+(k_{1}-1)\text{exp}(k_{1})]}{k_{2}^{2}-2k_{1}k_{2}+k_{1}^{2}}.$$ (13)

The logic can be extended to allow all the relative rates to be nonzero.

2. *x*_1,_*_k_* ≠ *x*_2,_*_k_*: *p*_branch_ = *p*(1 change) + *p*(2 changes)

*p*(*1* change): Examine the example *x*_1,_*_k_* = A, *x*_2,_*_k_* = T.

$$p\left(\text{1}\;\text{change}\right)=K_{k}r_{1}\left\{\exp\left[-2K_{k}\left(r_{1}+r_{2}+r_{3}\right)\right]+\exp\left[-2K_{k}\left(r_{1}+r_{4}+r_{5}\right)\right]\right\}$$ (14)

*p*(2 changes): Examine the example *x*_1,_*_k_* = A, *b*_2,_*_k_* = C.

Assume that only *r*_1_ and *r*_4_ are nonzero (Figure 2), and that A is the ancestral base and C is the derived base. The sequence of events is therefore an A → T change followed by a T → C change. The probability of this event sequence is obtained from:

$$\overset{1}{\underset{0}{\int }}p(\text{no mutation to time }y)\cdot p(\text{A}\to \text{T}\;\text{mutation})\cdot p(\text{T}\to \text{C}\;\text{mutation between time }y\text{ and}\;1)dy.$$ (15)

This is:

$$\frac{r_{1}r_{4}}{\left(r_{1}+r_{2}+r_{3})(r_{1}+r_{4}+r_{5}\right)}\overset{1}{\underset{0}{\int }}\text{exp}(-k_{1}y)k_{1}(1-y)k_{2}\times \text{exp}[-k_{2}(1-y)]dy,$$ (16)

where *k*_1_ and *k*_2_ have the same meanings as above.

The algorithm can be extended to cases where the relative rates are all nonzero.

### Computing uSFS elements

The ML approach described by Keightley *et al.* (2016) estimates the proportion of density, *π_j_*, attributable to the major allele being the ancestral allele *vs.* the major allele being the derived allele for each uSFS element pair (indexed by *j* and *m* – *j*, where *m* is the number of gene copies sampled). We implemented this algorithm as follows, conditional on the ML estimate of the rate parameters, $\hat{\phi }$ (obtained by evaluating Equation 1), which are therefore assumed to be known without error. For a uSFS containing *m* elements, *m*/2 ML estimates require to be made. Assuming sites evolve independently (*cf*. Equation 1), the likelihood of *π_j_* for the subset of sites (numbering sites*_j_*) having *j* copies of the minor allele in the focal species is:

$$L({\text{π}}_{j})=\prod_{i=1}^{{\text{sites}}_{j}}[p(y_{\mathrm{i,1}}|\hat{\phi }){\text{π}}_{j}+p(y_{\mathrm{i,2}}|\hat{\phi })(1-{\text{π}}_{j})],$$ (17)

where the probability of the observed nucleotide configuration for the focal species and the outgroups at the site is given by Equation 2, evaluated with the major allele $\left[p(y_{\mathrm{i,1}}|\hat{\phi })\right]$ and the minor allele $\left[p(y_{\mathrm{i,2}}|\hat{\phi })\right]$ as the state of the focal species at that site (see Figure 1).

### Computing ancestral state probabilities on a site-by-site basis

The probability of allele X*_i_ vs.* allele Y*_i_* being ancestral at site *i* could be computed from their relative probabilities, *i.e.*,$p_{1}=p(y_{\mathrm{i,1}}|\hat{\phi })\text{ and }p_{2}=p(y_{\mathrm{i,2}}|\hat{\phi }),$but this only uses information from the estimated rate parameters. It does not incorporate information from the number of major *vs.* minor copies at the site. For example, if the outgroup information were uninformative, we would assign *p*_1_ = *p*_2._ If there are few sites in the data set as a whole where the derived allele is at a high frequency, however, the estimated uSFS would tell us that A is more likely to be ancestral.

To infer the ancestral state probabilities for site *i*, information from the estimated rate parameters is augmented by the nearly independent information from the estimated uSFS (*cf*. Halligan *et al.* 2013). If there are *j* copies of the minor allele in the focal species at a site *i*, the probability of the major allele X*_i_* being ancestral is:

$$p({\text{X}}_{i}=\text{ancestral})=\frac{p_{1}\hat{{\text{π}}_{j}}}{p_{1}\hat{{\text{π}}_{j}}+p_{2}(1-\hat{{\text{π}}_{j}})}.$$ (18)

As a check on this equation, it can be shown that the sums of the ancestral state probabilities recovers the estimated uSFS.

### Simulations

We extended a simulation program described by Keightley *et al.* (2016) to simulate three outgroups for the topology illustrated in Figure 1. Briefly, unlinked sites with four nucleotide states were simulated in a diploid population of size *N* = 100. The mutation rate per site per generation was set to *μ* = *θ*/*N*, and the neutral genetic diversity, *θ*, was typically 0.01. The simulations allowed any variation within a population at a node of the phylogenetic tree to be passed to two ancestral subpopulations, which were formed by sampling chromosomes with replacement in one generation. To generate the data for uSFS inference, a single gene copy was randomly sampled from each outgroup species. We either simulated neutral sites, or a mixture of neutral and selectively constrained sites. If a mutation occurred at a selectively constrained site, its selection coefficient was *s*/2, where *s* is the difference in fitness between the homozygous mutant and the heterozygote. Fitness effects were multiplicative between and within loci.

### DPGP data

We analyzed fourfold degenerate sites from the Rwandan sequences of the DPGP phase 2 data, comprising 17 haploid genomes (see Keightley *et al.* 2016 for details).

### 1000 Genomes data

We downloaded variant calls from the phase 3 release of the 1000 Genomes Project (from ftp://ftp.1000genomes.ebi.ac.uk/vol1/ftp/release/20130502/) and extracted the 99 unrelated individuals from the Luhya in Webuye, Kenya (henceforth LWK) population. First, we restricted our analyses to sites that were fourfold degenerate in all autosomal transcripts of protein-coding genes in humans according to Ensembl release 71. We used the six-way EPO multiple alignments of primate species (available from ftp://ftp.ensembl.org/pub/release-71/emf/ensembl-compara/epo_6_primate/) to determine the alleles in orangutans and macaques at each fourfold degenerate site, and to determine whether those sites were within a CpG in humans or either of the outgroup species. We used orangutan and macaque as outgroups in our analysis. Chimpanzee and gorilla are closer and potentially more informative, but they share a high proportion of polymorphism with human and this violates an assumption of our analysis. The EPO multiple alignments were first converted from .emf format to .maf format, and then specific regions were accessed using the WGAbed package (https://henryjuho.github.io/WGAbed/). The data for the human ancestral alleles, as used by the 1000 Genomes Project (1000 Genomes Project Consortium 2015), were downloaded from ftp://ftp.ensembl.org/pub/release-74/fasta/ancestral_alleles/.

Sites were retained for analysis if there was no missing data in humans or either outgroup species. Sites were further assigned to CpG and non-CpG categories. CpG sites were defined as sites that were CpG in their context in any of the three species: human (including both REF and ALT alleles), orangutan, or macaque. Non-CpG sites were defined as sites that were never CpG in their context in any of the same species, including both REF and ALT alleles in the human sample. Alleles at polymorphic sites were used to populate the uSFS following two methods: (1) using the ancestral allele provided by the 1000 Genomes Project to polarize derived and ancestral variants, and (2) using the ML method described in the present study.

### Data availability statement

Software are available for download from https://sourceforge.net/projects/est-usfs/. Supplemental material available at Figshare: https://doi.org/10.25386/genetics.6275915.

## Results

### Simulation results

The uSFS inference method allows several outgroups to be included, but the extent of any benefit from additional outgroups has been unknown. To investigate this, we simulated unlinked sites according to the tree topology shown in Figure 1 with three outgroups, recorded the “true” uSFS, and compared it to uSFSs estimated using one, two, or three outgroups. High derived allele frequency uSFS elements are expected to be most affected by misinference (Baudry and Depaulis 2003; Keightley *et al.* 2016), so we focused on the last uSFS element (*e.g.*, element 19 of a 20-element uSFS). Our measures of bias and accuracy were the average deviation and root-mean-squared error (RMSE) for this element.

**Figure 3:**
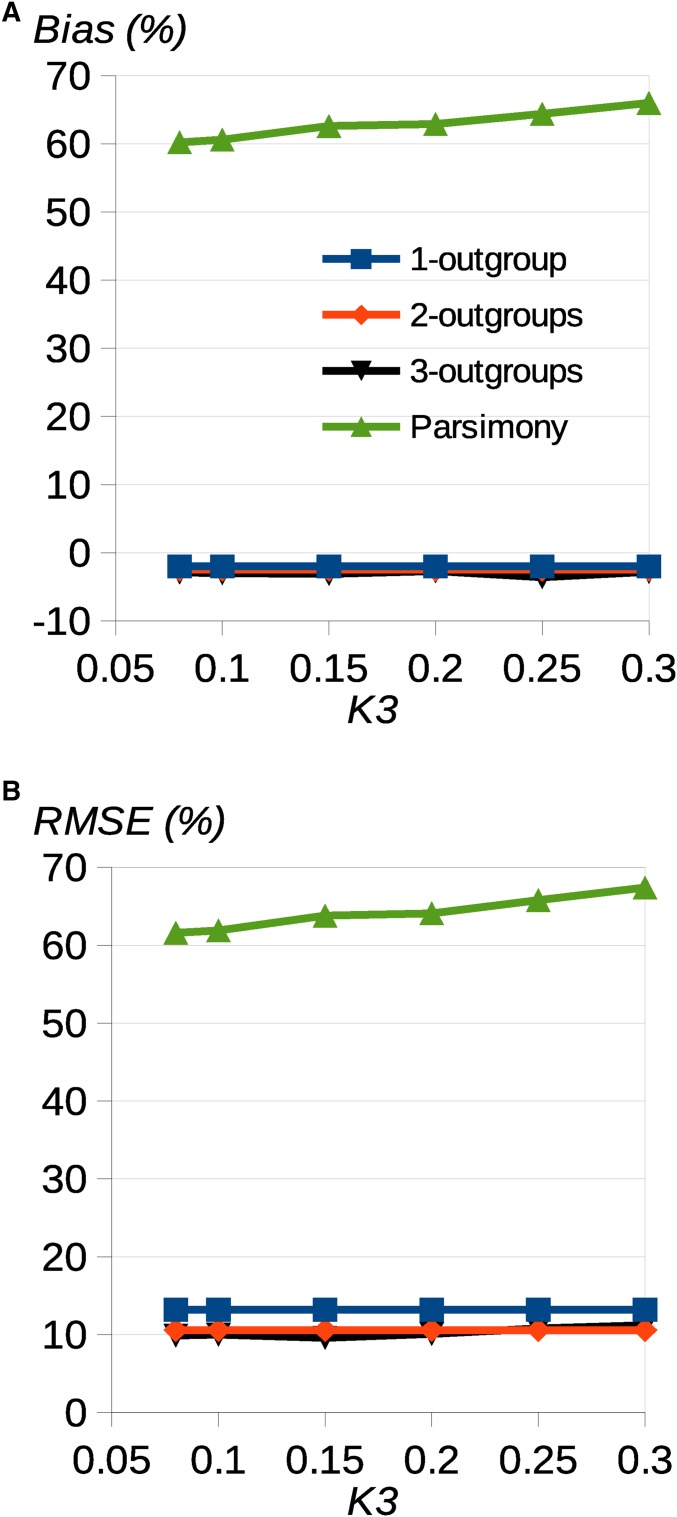
Effect of adding additional outgroups. Simulation results showing (A) the percentage bias = average deviation from the true uSFS, and (B) RMSE for uSFS element 19, as a function of divergence, *K*_3_, from a third outgroup. There were 100,000 sites simulated in 360 replicates under the JC model, and *K*_1_ = 0.1 and *K*_2_ = 0.1. There were 20 gene copies sampled at each site in the focal species. Blue, red, yellow, green = results from uSFS inference with one, two, and three outgroups and parsimony, respectively. Note that estimates for one and two outgroups are invariant as a function of *K*_3_.

For the case of neutrally evolving sites, if data are simulated and analyzed under the JC model, there is a small amount of negative bias affecting the last uSFS element (*i.e.*, high frequency uSFS elements tend to be slightly underestimated; Figure 3A). The cause of this negative bias is unknown, but it could be a consequence of violation of any of the assumptions described in the *Material and Methods*. RMSE is reduced somewhat if a second outgroup is added, but there is little benefit from adding a third outgroup (Figure 3B). If data are simulated including transition:transversion bias and the analysis is by the JC model, the last uSFS element is substantially overestimated (Supplemental Material, Figure S1). If the K2 or R6 models are used, however, only a small amount of bias is observed (Figure S1). As expected, parsimony-based inference seriously overestimates the frequency of high frequency derived alleles in all cases (Figure 3A and Figure S1). Parsimony does not provide ancestral state probabilities per site, because it assigns an allele as derived or ancestral with certainty. Parsimony will therefore be potentially seriously biased compared to computing ancestral state probabilities using Equation 18.

**Figure 4:**
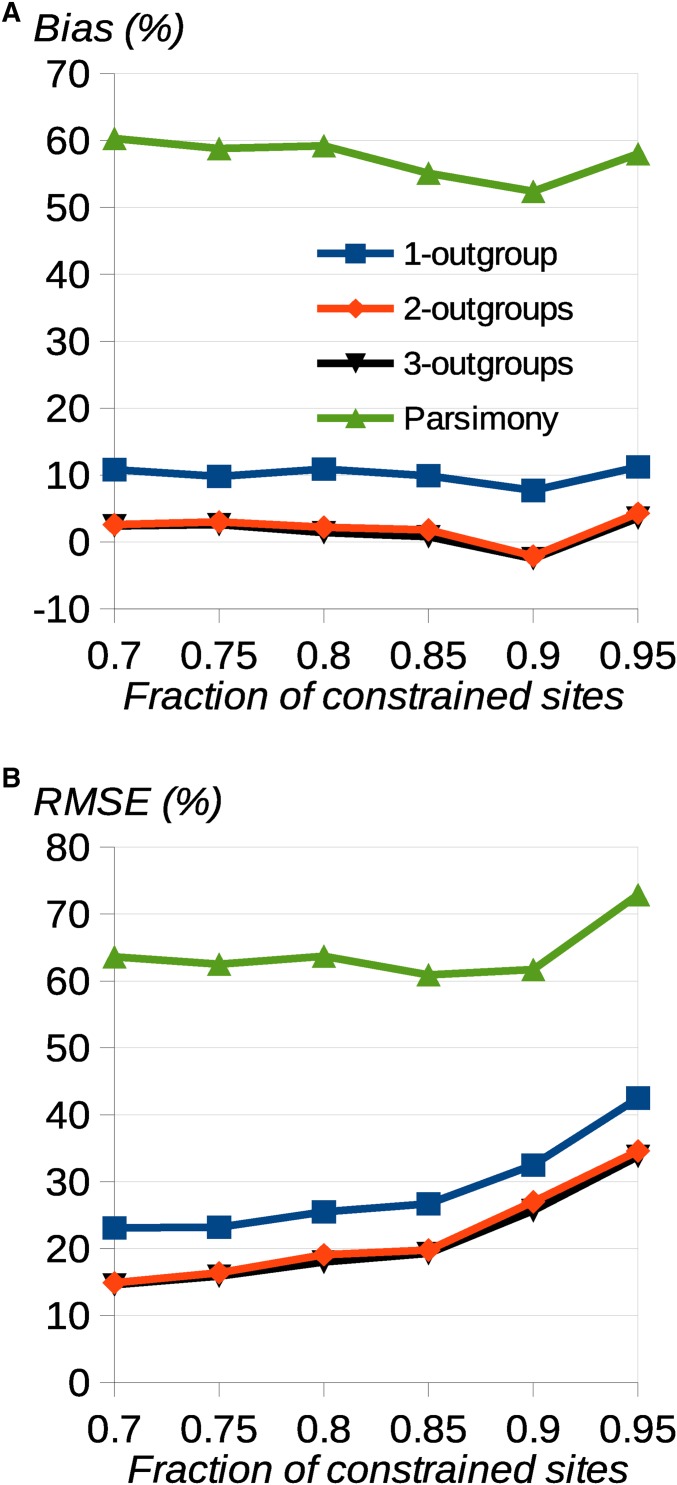
Effect of presence of selectively constrained sites on uSFS inference. Simulation results showing (A) the percentage bias and (B) RMSE for uSFS element 19 as a function of the fraction of constrained sites. There were 10,000 sites simulated in 3600 replicates under the JC model with three outgroups, and *K*_1_ = 0.1, *K*_2_ = 0.15, and *K*_3_ = 0.15. There were 20 gene copies sampled at each site in the focal species. Blue, red, yellow, green = results from uSFS inference with one, two, and three outgroups and parsimony, respectively.

We then investigated accuracy and bias for the case of a fraction of sites subject to moderate purifying selection (scaled selection strength *Ns* = 10). This is relevant for inferring the uSFS for nonneutral sites, such as nonsynonymous sites of protein-coding genes, and for cases where there is variation in the mutation rate among sites, leading to variation in the rate of substitution. Such variation violates an assumption of the uSFS inference method and is therefore expected to cause the method to break down to some extent. As we previously observed (Keightley *et al.* 2016), the presence of variation in the rate of substitution leads to overestimation of high derived allele frequency uSFS elements (Figure 4A). The bias can be serious if there is only one outgroup, but is reduced if a second outgroup is included. However, there is only a small additional benefit from adding a third outgroup. Variation about the observed values is lower, on average, if additional outgroups are included (*i.e.*, RMSE is lower; Figure 4B), but again adding a third outgroup is of little benefit. As expected, parsimony performs poorly, overestimating the high frequency derived allele frequency.

### Analysis of DPGP phase 2 data

To assess the performance of the uSFS inference procedure in a more realistic situation, we analyzed fourfold degenerate sites from the Rwandan sequences of the DPGP phase 2, which comprises 17 haploid genomes (provided by J. Campos). We compared the inferred uSFSs obtained using *Drosophila simulans* as the sole outgroup and using both *D. simulans* and *D. yakuba* as outgroups, and investigated the consequences of increasing the complexity of the substitution model. More complex substitution models fit the data much better (Table 1), largely driven by the approximately twofold transition:transversion mutation bias captured by the K2 model.

**Table 1:** Differences between log likelihood of simpler models and the R6 model

Model	Change in log likelihood	
	1000 Genomes	DPGP
JC	−35,000	−13,000
K2	−55	−1,400
R6	0	0

The log likelihoods are obtained from stage 1 of the analysis (estimation of rate parameters, see text). The data analyzed are fourfold degenerate sites from the1000 Genomes Project and Rwandan sequences of DPGP phase 2.

**Figure 5:**
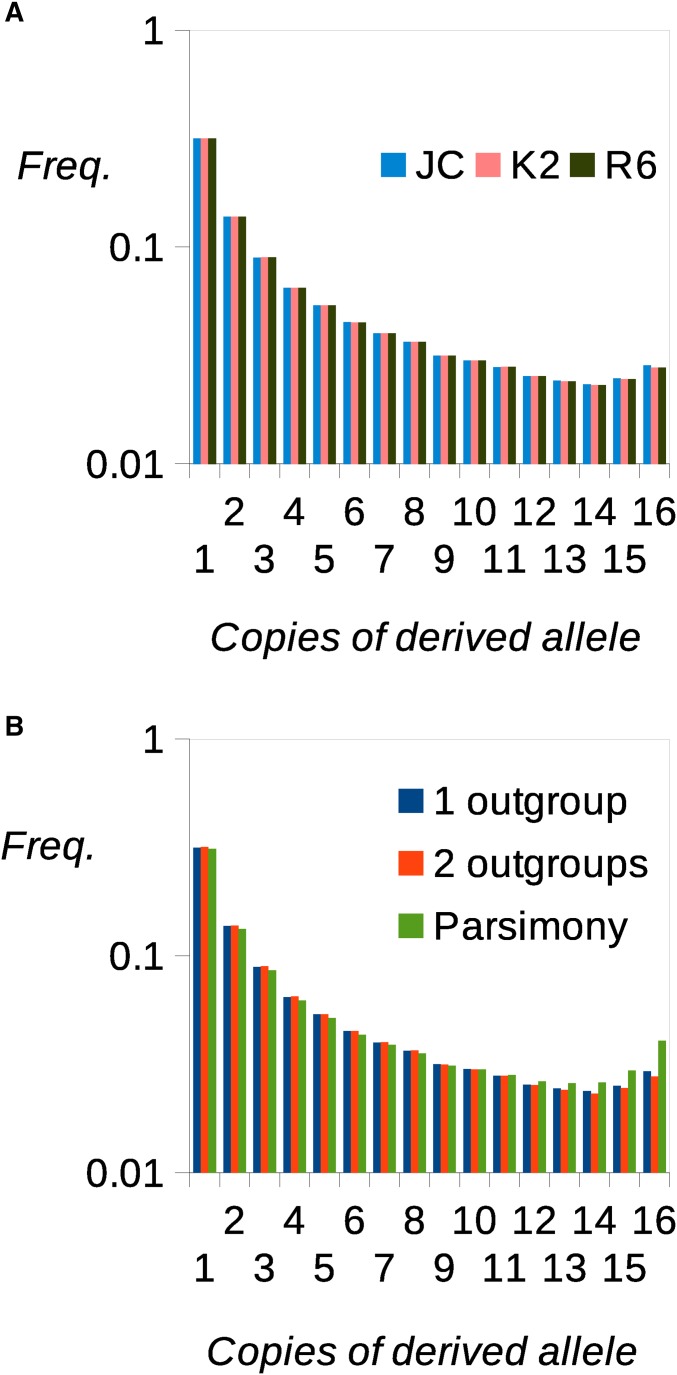
Analysis of fourfold degenerate sites of DPGP phase 2. (A) uSFSs estimated assuming three different substitution models. (B) uSFSs estimated using the method described in this article based on one outgroup (*D. simulans*) or two outgroups (*D. simulans* and *D. yakuba*) along with the uSFS inferred using parsimony. Freq., frequency.

Although different nucleotide substitution models produce large differences in log likelihood, the estimated uSFS is appreciably different only between the JC and K2 models, and it is indistinguishable between the K2 and R6 models (Figure 5A). Consistent with the simulation results, the inclusion of a second outgroup (*D. yakuba*) perceptibly reduced the high derived allele frequency uSFS elements, compared to using a single outgroup (*D. simulans*) (Figure 5B). There is an uptick at the right-hand side of the inferred uSFS, but it is unknown whether this is a consequence of misinference, ongoing positive selection on fourfold sites, or positive selection on linked sites. Consistent with the simulations, parsimony infers a substantially higher frequency of high frequency derived allele classes.

### Analysis of 1000 Genomes data

**Figure 6:**
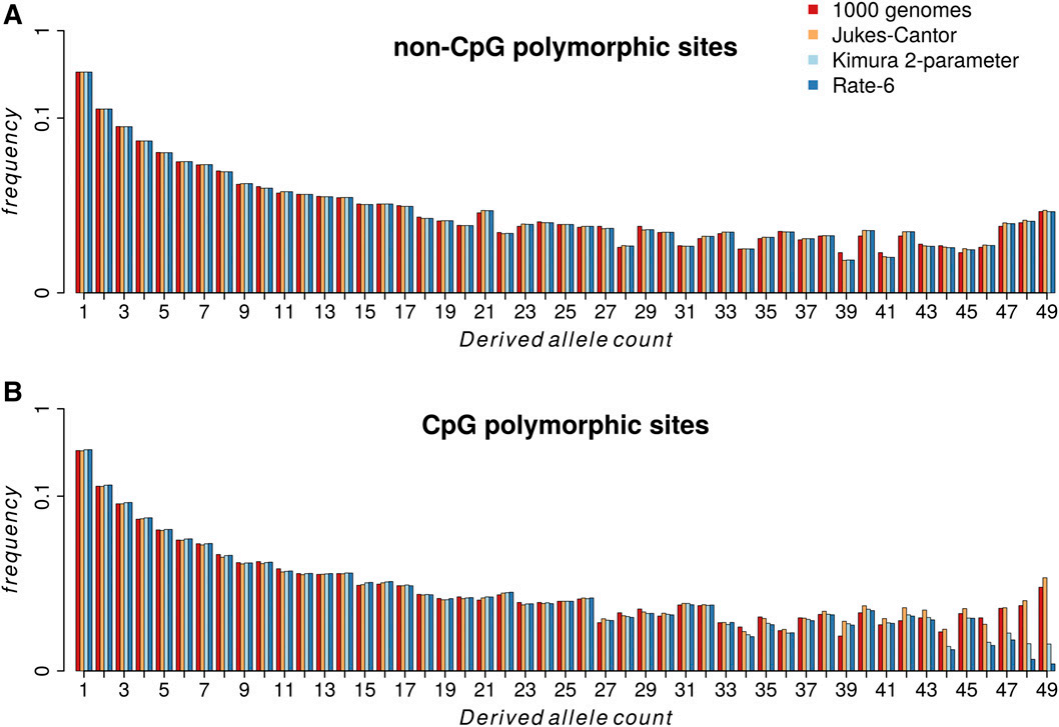
uSFSs for fourfold degenerate sites inferred by the 1000 Genomes Project and by the methods described in this article for three nucleotide substitution models. (A) non-CpG sites. (B) CpG sites.

SNP ancestral states inferred by the 1000 Genomes Project Consortium (2010, 2015) have been widely used (*e.g.*, Mondal *et al.* 2015; Yang and Slatkin 2016; Harris and Pritchard 2017). In their 2015 article, a heuristic approach was used to assign the ancestral state based on the inferred human–chimpanzee common ancestor and the human–chimpanzee–orangutan common ancestor. Allele frequency information was not incorporated. We reinferred the ancestral state at fourfold degenerate and zerofold degenerate sites in the LWK population, using the ML method presented here, and compared the resulting uSFSs (Figure 6 and Figure S2). Because uSFSs from the full data set of 99 individuals (198 chromosomes) were difficult to visualize, we downsampled the LWK population to 25 randomly chosen individuals. The results from the full data set are qualitatively similar to those from the downsampled data and are presented in Figure S3. uSFSs inferred using one or two outgroups show only minor differences (Figure S4).

For non-CpG sites, the uSFS produced using the 1000 Genomes Project’s ancestral states and the uSFSs produced by our ML method broadly agreed (Figure 6A). In contrast, for CpG sites, the results under the 1000 Genomes method and the JC model depart from the K2 model and the R6 model at the right-hand side of the inferred uSFS (Figure 6B). Under 1000 Genomes and JC, there is a pronounced uptick at high frequency derived variants, which is not present in the two more complex substitution models. In the case of the last uSFS element, for example, the 1000 Genomes and JC differ from the more complex models by about a factor of two.

CpG sites have an ∼10-fold higher mutation rate than non-CpG sites in humans, due to an elevation in the number of C → T and G → A transitions (Nachman and Crowell 2000). This was borne out in the inferred branch lengths and the ratio of transition rate to transversion rate (*κ*) at the two classes of site. Under the R6 model, which is the best-fitting model for both classes of site, the length of the branch between the human–orangutan common ancestor and humans was 0.0083 for non-CpG sites and 0.092 for CpG sites. Estimates of *κ* under the K2 model were 4.2 and 8.3 for non-CpG and CpG sites, respectively, which are broadly in agreement with previous studies (*e.g.*, Keightley *et al.* 2011).

## Discussion

This article generalizes a method we previously developed for inferring the uSFS (Keightley *et al.* 2016) by allowing the inclusion of multiple outgroup species and potentially any phylogenetic tree topology (although only topologies of the type illustrated in Figure 1 have been implemented in the software). The new method gives nearly identical results to the previous method if the same outgroups are analyzed and the same substitution model is assumed. The new method implements three substitution models: the JC, K2, and R6 models. These models are nested. The K2 model gives the same likelihood as the JC model if the transition:transversion ratio parameter *κ* is fixed at 1. If the R6 parameters are constrained such that *r*_3_ = *r*_4_ (transition mutations) and *r*_1_ = *r*_2_ = *r*_5_ = *r*_6_ (transversion mutations) (see Figure 2), the same ML is obtained as the K2 model. Consistent with our previous results (Keightley *et al.* 2016), simulations suggest that the inclusion of a second outgroup generally increases the accuracy of uSFS inference, especially in the presence of variation in the rate of substitution among sites. The inclusion of a third outgroup did not, however, lead to a further improvement in uSFS inference accuracy. In the real data sets we have analyzed from *Drosophila* and humans, more complex substitution models gave higher log likelihoods in stage 1 of the analysis (evolutionary rate parameter estimation; Table 1), but this did not translate into a benefit in stage 2 (uSFS element inference) beyond the K2 model. The nucleotide substitutions models implemented are somewhat simplified in the sense that rates of change between pairs of nucleotides are symmetrical and these parameters do not vary between branches. It is possible that more complex models allowing these complications would lead to a further improvement, given that such effects are common in real data. A further weakness we hope to address in the future is its noncontext dependence of a substitution model (so we cannot deal with hypermutable CpGs), and further development along the lines of, for example, Arndt *et al.* (2003) will be needed.

We investigated whether our new method and parsimony produce different results when applied to real data. In the case of DPGP phase 2, parsimony estimates a much higher proportion of high frequency derived alleles (Figure 5). This has consequences for population genetic analysis. For example, if a three-epoch demographic model is fitted to the fourfold SFSs estimated by parsimony and by our present method (Schneider *et al.* 2011), the inferred population size changes and timings differ substantially (Table S1). In the case of the 1000 Genomes Project, we divided the data into CpG and non-CpG sites and inferred uSFSs separately for each class. At non-CpG sites there was a close agreement between the uSFS generated using the 1000 Genomes Project’s ancestral alleles to polarize variants and the uSFSs generated using the ML method. Parsimony is a more justifiable method of reconstructing ancestral states when the amount of change is small over the evolutionary time being considered, because it assumes *a priori* that change is unlikely (Felsenstein 1981). In contrast, parsimony is likely to be less accurate at CpG sites, which have an ∼10-fold higher rate of evolution. Our results bear this out. The uSFSs for CpG sites differed in the frequency of high frequency derived variants between the 1000 Genomes and the K2 and R6 models by up to a factor of eight. These are the class of variants where the greatest probability of misinference is expected. The JC model more closely mirrored the 1000 Genomes Project uSFS, presumably because it was unable to capture the ratio between transition rate and transversion rate at CpG sites, which is around twofold more extreme compared to non-CpG sites.

**Figure 7:**
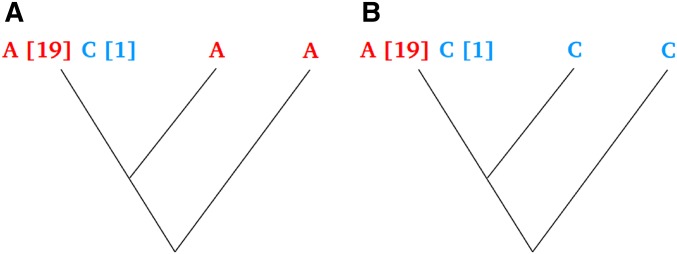
Example of a polymorphic site where 20 gene copies are sampled in a focal species and two outgroups have different nucleotide states. (A) Major allele agrees with outgroups. (B) Minor allele agrees with outgroups.

We have also addressed the problem of calculating ancestral state probabilities for polymorphic sites on a site-by-site basis. In doing so, we take into account both the nucleotide substitution parameter estimates (which determine the frequencies of multiple hits) and the frequencies of derived *vs.* ancestral alleles at other sites in the data. There are two main situations where this can make a significant difference compared to using parsimony. The first concerns sites where the outgroups are different in state from the focal species. These sites are frequently removed from the analysis (*e.g.*, Keinan *et al.* 2007; Sabeti *et al.* 2007; 1000 Genomes Project Consortium 2010, 2015; Langley *et al.* 2012), leading to underrepresentation of polymorphic sites, especially sites that have a low frequency of the derived allele, which tend to be the most common. The second situation concerns the tendency of parsimony to overestimate the frequency of sites with a high frequency of the derived allele. Consider the two configurations of nucleotides at a site of focal species and two outgroups shown in Figure 7. Assume that this is one of a large number of sites generated by simulation. At the site in question, there are 19 As and 1 C in the 20 gene copies sampled. In Figure 7A, the two outgroups are state A. By parsimony, the ancestral allele of the variation in the focal species would therefore be assigned as A. If the branch length b_1_ (Figure 1) is 0.05, and using only information from the inferred substitution rates (*i.e.*, using the relative values of *p*_1_ and *p*_2_ calculated using Equation 2), *p*(A = ancestral) = 0.98. Taking into account the fact that high frequency derived allele sites are rare in the data set as a whole, and applying Equation 18, base A is even more strongly supported as the ancestral allele, *i.e.*, *p*(A = ancestral) > 0.99. This illustrates that parsimony is a good approximation for sites likely to have a low number of derived gene copies. The outcome is different for Figure 7B, where the two outgroups have the same state as the minor allele of the focal species. By parsimony, the ancestral allele would be assigned C, implying that we are certain the site has 19 copies of the derived allele. Using only information from substitution rate parameters and applying Equation 2, *p*(A = ancestral) = 0.016. Taking into account other sites in the data, which tell us that sites having 19 derived allele copies are uncommon, and applying Equation 18, *p*(A = ancestral) = 0.14. Thus, we are much less certain that the derived allele is A at this site. This probability increases (decreases) if the outgroups are more distant (closer) to the focal species.
